# Understanding biopharmaceutical investment decision-making: how does Congressional Budget Office's model compare to investor insights?

**DOI:** 10.1093/haschl/qxaf200

**Published:** 2025-10-23

**Authors:** Matthias P Hofer, Priscila Radu, Mikel Berdud, Amanda Cole, Graham Cookson

**Affiliations:** Office of Health Economics, London SE1 2HB, United Kingdom; Office of Health Economics, London SE1 2HB, United Kingdom; Office of Health Economics, London SE1 2HB, United Kingdom; Office of Health Economics, London SE1 2HB, United Kingdom; Office of Health Economics, London SE1 2HB, United Kingdom

**Keywords:** IRA, CBO, innovation, pharmaceuticals, investors, investment

## Abstract

The Congressional Budget Office (CBO) created a model of new drug development with the aim of informing the US Congress about the potential impact of policy changes affecting expected biopharmaceutical revenue on future innovation. While models are by their nature simplifications of reality, biopharmaceutical investment decision-making is particularly complex and poorly understood outside the investment ecosystem, raising questions about the adequacy of such models to inform policymaking. To better understand how CBO's model compares to real-world investment decision processes, we conducted semi-structured interviews with investors representing venture capital, private equity, corporate venture capital, and biopharmaceutical companies. The interviews with investors suggest that the CBO's model does not adequately reflect investment decisions for drug development. These findings highlight the risks of using models to guide policymaking and the need to improve the existing model with the help of stakeholder input before such models are adopted.

## Teaser

As US policymakers consider new pharmaceutical pricing reforms, understanding how such policies influence investment in drug development is critical. This study compares the US Congressional Budget Office's (CBO) model of drug development with insights from biopharmaceutical investors—including venture capital, private equity, and corporate venture capital stakeholders. Through qualitative interviews, we identify key discrepancies between the model's assumptions and the complex realities of investment decision-making. Investors described firm-level investment strategies and innovation dynamics that do not accord with the CBO model structure and assumptions, and also referred to the impact of recent policy changes such as the Inflation Reduction Act. Our findings highlight the need for modeling efforts to be improved to better reflect real-world investment decision-making when forecasting innovation impacts and guiding policy design.

Key Points

- A wide range of biopharma investors affirm that the Congressional Budget Office (CBO) model does not adequately capture the realities of drug development investment decision-making. Specifically, investors identified several oversimplified assumptions related to the diversity and complexity of the investment ecosystem, the nature of how investment decisions are made at the product and firm level, and how innovation is even measured.
- In contrast to the modest innovation impacts forecasted by CBO, the Inflation Reduction Act (IRA) has already influenced investor behavior, steering investments from certain biopharma sectors.
- As new pharmaceutical pricing policies emerge, it is essential that the CBO model be continuously improved through rigorous research and inclusive stakeholder input. Further, there should be clear and transparent dialogue around what can and cannot be captured by such modeling efforts.

## Introduction

Pharmaceutical innovation has been shown to improve both patient survival and quality of life, as well as to enhance healthcare system performance.^1,2^ While pharmaceutical research and development (R&D) is a global enterprise, the United States (US) conducts more pharmaceutical research than any other country^3^ and provides broader and more rapid access to new medicines.^4^ These factors contribute to the US having the largest pharmaceutical market globally.^3,5^

Drug pricing has become an increasingly prominent policy issue in the US, prompting a series of legislative initiatives aimed at reducing costs. These include the Inflation Reduction Act (IRA),^6^ which introduced the Medicare Drug Price Negotiation Program in 2022, and the proposed most-favored-nation pricing policy, a form of international reference pricing.^7^

While lower drug prices can benefit patients and health systems through improved affordability, reduced revenues can lead to weakened incentives for pharmaceutical R&D investment^8,9^ and fewer new innovations.^8,10,11^ To appraise this tradeoff, there is a need to understand the empirical relationship between biopharmaceutical revenues and innovation. However, this relationship is notoriously difficult to measure, with estimates in the published literature of the “elasticity of innovation” (ie, the % change in innovation relative to the % change in expected revenue) ranging between 0.23 and 6^10-17^ Furthermore, many empirical estimates are of limited relevance due to their age and the evolving nature of pharmaceutical innovation. In a previous Delphi study, no consensus could be reached on the most appropriate magnitude of this relationship.^18^ Furthermore, there was agreement among respondents of that Delphi study that a single point estimate would not be reflective of the fact that the relationship will vary by firm size and type, therapeutic area, as well as the size of the market shock (ie, how radical the policy change).

The Congressional Budget Office (CBO) is tasked with predicting the effects of any US policy that alters the expected costs of returns from new drug development. CBO's approach has evolved over time, with different versions from 2019, 2021, and 2022. More recently, the CBO announced another model update which incorporates some differentiation by drug type; dependencies across drug development stages; and some new sources of data.^19^ In applying their model, the CBO predicted that the IRA could reduce global revenues from sales of new drugs by 1% to 3%, with approximately a 1% reduction in innovation (equating to a modest 13 fewer drugs over the next 30 years).^20^ Projections of this nature have been hotly debated in the literature; the CBO's overall finding of modest projected IRA impact had been supported by some^21-23^ and contested by others.^24,25^

Acknowledging the inherent uncertainties and limitations, the CBO has issued multiple calls for research to improve the methodology and data used in their model. The objective of this study was to contribute to this discussion by characterizing investor decision-making within the life sciences innovation ecosystem from the perspective of a diverse range of biopharma investors. By organizing interview insights against the key assumptions of the CBO model, we hope to shed light on how well the reality of the existing innovation ecosystem is represented in the assumed reality of the model (Table 1).

**Table 1.** Contrasting four key model assumptions by CBO with investment reality.

CBO model assumption	Investor & industry reality
**1. One homogeneous innovator and investment firm with product-level investment decisions can represent the R&D ecosystem.**	The biopharma ecosystem is diverse and complex, involving a range of actors (small and large companies, VCs, corporate investors). Small companies often drive early R&D and rely on external funding, while large companies engage in M&A and partnerships. Investment decisions are typically made on a company's full portfolio rather than individual product.
**2. Firm characteristics and risk tolerance preferences are irrelevant to decision-making.**	Investment decisions vary by company size, development stage, and investor type. Further, R&D costs are rising due to growing complexity and trial logistics, affecting companies differently. Smaller firms adjust strategies based on cost and feasibility, often targeting unmet needs or planning early exits. They build portfolios to attract acquisition. Larger firms and investors increasingly prioritize time-to-market and trial efficiency. They balance in-house and external investments. Early-stage VCs accept higher risk and diversify across assets. Late-stage VCs focus on operational risks and market access.
**3. Investments are made using a simple decision rule that only a nominal return on investment is needed.**	While financial calculations like net present value (NPV) is a factor to support investment decisions, it is not sufficient on its own. Especially early-stage investors rely less on NPV and more on strategic fit and potential. They especially require high ROI thresholds (eg, three times or more) due to risk. Biopharma firms consider multiple factors including unmet need, therapeutic area, and regulatory landscape in investment decisions.
**4. The number of newly approved drugs is an adequate measure of innovation.**	Innovation extends beyond initial approvals to include new and additional indications and patient populations thereafter. Investors warn that the IRA's design may discourage post-approval R&D, especially in oncology, rare diseases, and Medicare-relevant areas, due to distorted incentives and pricing timelines. Beyond the impact on number of drugs approved, a measure of their health benefit would offer a more meaningful picture of the impact of policy change.

## Methods

Semi-structured interviews were conducted with a wide range of investors: seven venture capital investors (VCs), who typically engage in early-stage funding and provide strategic guidance to emerging biopharma companies; three corporate venture capital investors (CVC), who represent the investment arms of established pharmaceutical companies; two private equity investors (PE), known for their involvement in later-stage investments; four small biopharmaceutical companies, which are often at the forefront of R&D efforts but can face challenges in securing funding; and three large biopharmaceutical companies, which possess more extensive resources and infrastructure. A total of nineteen semi-structured interviews were conducted by two analysts between June and August 2024 (Supplementary material). Interviews were recorded, transcribed, and thematically coded. Data were analyzed using an analytic framework that encompassed six domains: 1) characterization of the innovation ecosystem; 2) investment type (company vs product investment); 3) risk and capital mobility; 4) investment decision-making; 5) costs of R&D; and 6) impact of IRA. The semi-structured interview format contained a mixture of closed and open-ended questions, allowing participants to express their opinions on the current investment environment, describe their investment strategies, discuss information relating to key assumptions of the CBO model, and offer viewpoints regarding the impact of policy changes associated with the IRA (Supplementary material). All interviewees were familiar with IRA, the innovation ecosystem, and their own investment decision context. While questions were designed to elicit information relevant to the appropriateness of the CBO model's key assumptions, the CBO model itself and its specific assumptions and parameters did not form the sole explicit focus of our questioning.

The interviews were supplemented with a targeted literature review (TLR) to characterize the innovation ecosystem landscape. The TLR was designed to gather a collection of impactful, thematically relevant sources to guide a focused synthesis of findings, using Google search engine as the primary search tool due to its wide accessibility and utility in capturing both academic and gray literature. This strategy provided a structured yet flexible framework to highlight the most relevant literature. For the illustrative example of the innovation history of ibrutinib (Imbruvica), we sourced background data from Pitchbook.^26^ Additionally, data on drug development pipeline and authorizations were collected through Citeline's PharmaProjects database between 2019 and 2024 to provide evidence of the types of investors and stakeholders involved in R&D and medicines approval.^27^

### Key CBO assumption #1: one homogeneous innovator and investment firm with product-level investment decisions can represent the R&D ecosystem

**Figure 1.**
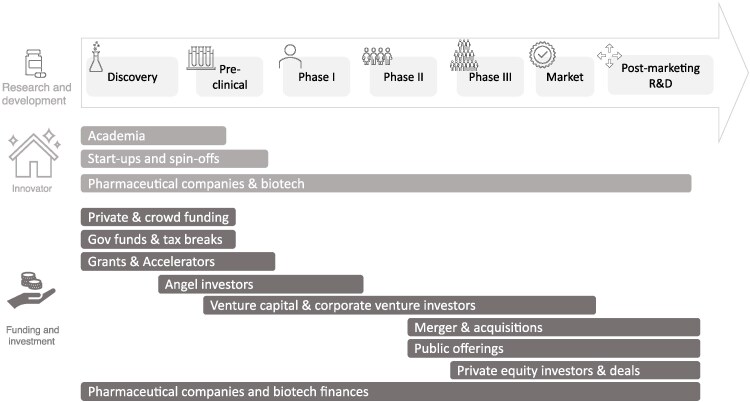
A graphical representation of the innovation investment ecosystem, adapted from^28,29^

The CBO model assumes that there is a single, homogeneous company representative of the entire industry, making product-level investment decisions. This mischaracterizes the more complex and diverse innovation and investment ecosystem that exists today and is depicted by Figure 1.

The biopharmaceutical ecosystem consists of both innovators—entities that develop and launch new therapies—and investors who provide the capital necessary to support R&D. These roles often overlap, with some actors functioning as both innovators and investors. In the very early stages, support typically comes from private and crowd funding, government grants, tax incentives, and accelerator programs. As R&D projects progress, angel investors and venture capital—both independent and corporate—play an increasingly prominent role, particularly through Phase II of clinical development. In the later stages, financing is predominantly provided by pharmaceutical and biotech companies themselves, supplemented by mechanisms such as mergers and acquisitions, public offerings, and private equity investments.

**Figure 2.**
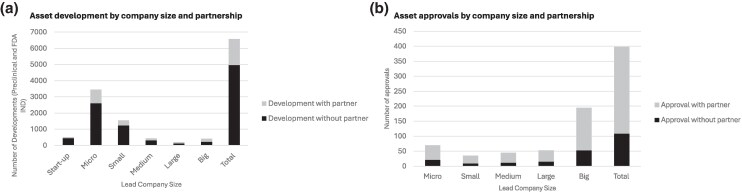
The development (A) and approval (B) of medicines between 2019 and 2024.^27^ The development figure includes development at preclinical and early clinical/IND stages. Definitions: Micro-size company: companies with market cap below $500 million; Small-size company: companies with market cap between $500 million and $2 billion; Mid-size company: companies with market cap between $2 billion and $10 billion; Large-size company: companies with market cap between $10 billion and $30 billion; Big-Pharma company: companies with market cap between $10 billion and $30 billion; Private companies do not publish market cap data. To avoid they are considered micro by default we have done annual searches to classify them by size.

On the innovator side, data show that preclinical and early clinical development is driven by smaller companies on their own, but that over time the level of cooperation between innovators becomes critical to bring a product to market as most assets (∼70%) are being developed and launched in partnerships (Figure 2).^27^ Our interviews confirmed that large Biopharma companies tend to focus on product-level investment (eg, in-house and licensing) with additional merger and acquisition (M&A) activities. While small Biopharma companies tend to focus on product-level R&D, they require buy-in from investors throughout the asset progression pathway. On the funding side, our interviews revealed that financial investors are primarily focused on company-wide investments rather than on specific product portfolios.

**Figure 3.**
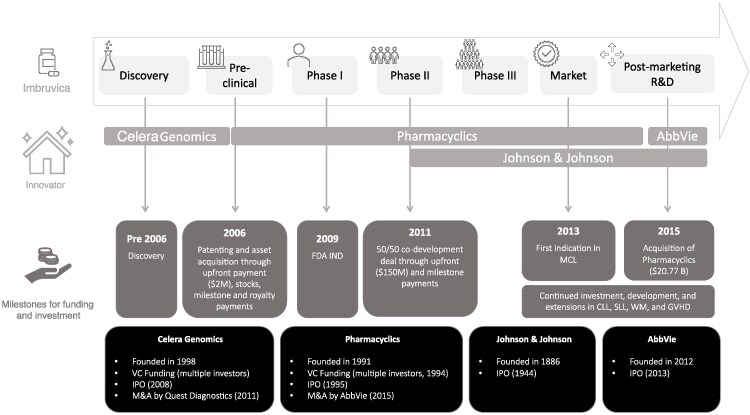
Overview of the innovation ecosystem that underpins the development of ibrutinib (Imbruvica). Synthesized from information from.^26,30,31,34^ Disease abbreviations: MCL: Mantle Cell Lymphoma; CLL: Chronic Lymphocytic Leukemia; SLL: Small Lymphocytic Lymphoma; WM: Waldenström's Macroglobulinemia; GVHD: Graft-vs-Host Disease.

An example of the ecosystem's complexity and the pivotal importance of all players is the development of ibrutinib (Imbruvica) (Figure 3). The molecule was initially discovered by the small biotechnology firm Celera Genomics^30^ and subsequently acquired and patented by Pharmacyclics^30-32^ Pharmacyclics advanced the asset to Phase II clinical trials supported by venture capital investment, public market financing, and strategic mergers and acquisitions,^33^ before entering a co-development agreement with Johnson & Johnson during Phase II.^34,35^ Following the acquisition of Pharmacyclics by AbbVie,^36,37^ the development and commercialization of ibrutinib were jointly undertaken by AbbVie and Johnson & Johnson. These larger pharmaceutical companies leveraged their scientific expertise and financial resources—including internal capital, debt instruments, and equity financing—to bring the therapy to market and subsequently continue its development to support indication expansions to reach new patient populations.^37,38^

The CBO model conceives a single decision-maker making product-level decisions. As demonstrated in this section, this masks the complexity of investment decisions particularly in the early phases, where R&D is driven by small companies, enabled by investors who make decisions at the portfolio- rather than asset-level.

As a result, the current CBO model is limited in its capacity to capture the diverse types and magnitudes of impacts across key stakeholders within the innovation ecosystem. This limitation may lead to the masking of heterogeneity in model parameters and in stakeholder responses to various policy interventions. The direction and extent of any resulting bias in model output estimates will depend on the specific policy context and its differential effects on the actors involved.

### Key CBO assumption #2: firm characteristics and risk tolerance preferences are irrelevant to decision-making

The CBO model makes several simplifying assumptions that limit its applicability to real-world pharmaceutical investment decisions. It assumes homogeneity across firms—ignoring differences in size, development costs, strategic priorities, and investor profiles—treating all actors as if they operate under identical conditions. It evaluates investments on a product-by-product basis, overlooking the portfolio-based strategies that companies and investors typically employ. Whereas in previous iterations of CBO's model, signals about a drug candidate's likelihood of success are unrealistically assumed to be independent across phases of development, the latest update allows signals of a drug's revenues and costs to be correlated across decision points.^19^ This represents a methodological improvement, though the data informing these success signals appear somewhat limited.

In practice, firms and investors dynamically assess and manage risk over time, adjusting investment decisions based on evolving evidence, financial capacity, and strategic considerations such as asset diversification. Our interviews revealed that risk tolerance and R&D investment decisions vary significantly by investor type and development stage.

For small biopharma companies, risk tolerance was reported to be highest in early clinical phases, where portfolios are often constructed to appeal to potential acquirers. These firms aim to demonstrate early promise in areas of unmet need, anticipating acquisition before the onset of costly late-stage trials. In contrast, large biopharma companies shared that they generally adopt a more balanced approach, managing risk through a mix of in-house development and acquisitions—often via corporate venture capital arms—with a strong focus on avoiding late-stage failures where sunk costs are greatest.

Venture capital (VC) investors exhibit distinct patterns as well. Early-stage VCs tend to accept higher technical risk, mitigating it by diversifying across multiple companies, technologies, and therapeutic areas. Late-stage VCs, while facing less scientific uncertainty, must navigate operational risks such as manufacturing, supply chain logistics, and market access challenges.

Insights into these differentiated strategies also underscored the limitations of the CBO's ceteris paribus assumption—that all other factors remain constant. In practice, interviews reported rising R&D costs, driven by increasing complexity in drug development and clinical trial logistics. They confirmed that firms are affected by such rising costs unevenly. Smaller companies, in particular, struggle to finance full development independently and often tailor their strategies to secure early returns through partnerships or exits. VC investors supporting these firms are increasingly involved in optimizing early-stage efficiency and cost management. Larger firms, meanwhile, place growing emphasis on time-to-market and trial duration when evaluating investment opportunities.

The diverse actors and their decision-making remits, including risk tolerance, could be better reflected by a model that adjusts for these with differential stage-specific decision criteria.

### Key CBO assumption #3: investments are made using a simple decision rule that any return justifies investment

The CBO's model of pharmaceutical decision-making assumes that a positive net present value (NPV) is the sole basis for investment decisions. While all interviewees agreed that the predicted future revenue stream fundamentally determines the level of investment, they emphasized that any positive NPV is *not* the sole or sufficient basis for investment decisions. In contrast, bigger bets in light of technical uncertainty require bigger returns and one size does not fit all investment decisions.

Financial investors explained that they invest in companies and use internal modeling tools to support investment decisions, but that NPV calculations or revenue estimates are not generally considered reliable at early stages of investment. Importantly, while NPV calculations can play a larger role in later stages of development, financial investors require a much higher expected return on investment (ROI) at the time of exit (when they sell their ownership), given the technical uncertainty and high risk associated with investments. Investors tend to have different expectations for ROIs depending on the time of investment and risk taken, but most generally require a benchmark of three times returns or higher to make investments worthwhile according to investors interviewed.

Biopharma company interviewees explained that while they weigh the potential ROI through revenue from sales of the product once approved in their investment decisions and risk calculations, they also consider a multitude of additional factors that impact decisions to invest, including level of unmet need, preliminary evidence, therapeutic area, type of asset and regulatory requirements. Investment appraisals, and therefore relationships between market size and R&D, vary across different therapeutic areas and modes of action.

Interviewees discussed this in relation to the impact of IRA, which they are actively taking into account in their investment decision-making, potentially scaling-back investment in high-risk high-reward innovation such as those technologies that address a high existing unmet need. Particularly small biopharma interviewees highlighted this concern as they are highly dependent on external capital for R&D investment. Larger biopharma company interviewees confirmed that their internal capital is generally less mobile, but their ability to acquire products developed with early-stage VC funding would be harmed if capital shifts away.

Because the CBO model assumes any positive NPV leads to investment, it likely overestimates the number of drug candidates advancing, thereby underestimating the R&D output effect of a policy that curtails revenue. The CBO model could be amended by applying a higher cost of capital, particularly to the early development phases where capital is highly mobile and seeks the highest returns. In this context, investors warned that more competitive markets, such as AI and tech, are already being considered as more promising alternatives to the biotech industry.

### Key CBO assumption #4: the number of newly approved drugs is an adequate measure of innovation

The CBO's modeling only estimates the effect on the number of novel drug approvals, which ignores approvals for new indications of existing drugs (eg, in different diseases or sub-populations). Today, a significant proportion of treatment approvals are for indications following initial FDA approval: as high as 75% for targeted oncology treatments.^39^

Investors highlighted that only considering impact on the number of newly approved drugs, while omitting impact on new indications, is a clear example of how the model's output fails to capture the full impact of a policy that impacts revenue. This is especially true of a policy like the IRA which impacts revenues late in the product lifecycle, which will negatively influence post-approval research and development of new indications. They warned that this is likely to lead to more strategic decision-making around the sequencing of (or decision to launch) new indications. They also believe that the IRA is likely to have a disproportionate impact on certain therapeutic areas, such as rare disease (for which treatment advances often arise from post-approval research), oncology (which also benefits significantly from post-approval research, and which is dominated by small molecules, which are subject to earlier price negotiation compared with biologics), and therapeutic areas affecting the population covered by Medicare.

CBO's model would be improved by incorporating the impact of policy change on post-approval research, as not doing so is likely to underestimate the innovation impact. This effect may be mediated by the type of policy change and when in the lifecycle of a medicine that impact is realized. Further, by considering only the number of new drugs, the model does not attempt to capture the health impact of those. This would be a more meaningful measure of innovation and would enable a more comprehensive understanding of the type of drugs/patients most impacted by the policy change, and the resulting impact on population health.

## Discussion

Outside the US, healthcare systems globally are increasingly employing diverse strategies to negotiate and regulate the pricing of pharmaceutical innovations.^40,41^ These strategies have been shown to significantly reduce pharmaceutical revenues over time.^41^ In the US, there are comparatively lower levels of federal price regulations and the market has historically allowed manufacturers to set launch prices with relatively little regulation.^42^ The IRA marked a shift away from this system and the most recent announcements regarding international reference pricing further consolidate this shift^42-44^

The exact impact of new pricing regulation and setting in the US are subject to ongoing debates, especially considering the country's role as the largest pharmaceutical market and a focal point for global pharmaceutical research.^3,5,41^ In this context, it is crucial that the CBO model is evidence-based and closely reflects real-world conditions to adequately project both intended and unintended consequences of such a significant policy change.^45^ Results from this qualitative interview series with various types of investors can help support an enhanced understanding of that environment.

Our research reveals that the innovation ecosystem and investment decision-making is substantially more complex than the assumptions in the CBO models (Table 1). It features diverse sources of investment across the lifecycle of a product, differing in risk, capital, and other factors that affect investment decisions. Previous studies on biopharma investment also confirm that financial investors are a crucial part of the innovation ecosystem,^46,47^ and so understanding the role that they play is crucial.

Our research indicates that R&D costs in the biopharmaceutical industry are rising, suggesting that the CBO model may understate impacts by using historical estimates of R&D costs. The scientific literature supports these reflections by investors, identifying increasing R&D costs over time.^48,49^ Furthermore, our research highlights that the CBO's assumption that any NPV will generally be considered a positive investment signal is incorrect. R&D into pharmaceutical innovation requires adequate returns and one size does not fit all investment decisions. This is exemplified by a recent publication where NPV calculations were simulated across different investment decision time points, showing that NPVs at early stages of development are more significantly impacted by policy changes like the IRA.^50^

Our research also highlights that focusing the study of policy impact solely on the number of medicines developed misses important details, and that policy design has consequences on the type of innovation that is de-prioritized. For example, early empirical research since the implementation of the IRA has found post-approval industry-funded clinical trials for small molecule drugs declined by 47% since the IRA became law,^51^ and that early-stage biotech investment in small-molecule development dropped 70% after the law was introduced.^52^ These results exemplify the impact of the IRA on post-approval research and the disproportionate impact on small molecules, which would not be captured in CBO's model outputs.

While substantial evidence on the long-term impact of the IRA is still lacking, existing studies provide varied estimates. Some suggest positive effects of IRA on the development and investment in new innovations,^53^ others indicate no notable effect,^21-23,54^ and many predict negative effects^22,50-52,55-59^ In particular, there remains a lack of clear evidence on IRA's impact on financial investment in the biopharma sector as a whole,^22,23,59^ while there is evidence and growing investor consensus indicating a decline in investment in specific types of medicines, such as small molecules.^52,59^ Our research contributes to this debate by moving beyond the interpretation or projection of interim evidence. It captures the current perspectives of a wide range of investors, who agree that financial and R&D investment is mobile and that the introduction of policies that impact revenue, such as the IRA, has already influenced their own investor behavior, steering investments away from certain biopharma investments.

## Conclusion

Modeling the effects of policy on pharmaceutical innovation is a central component of ongoing policy discussions. Given the influence these models can have on shaping drug pricing policy, it is essential that their assumptions, methodologies, and implications are critically evaluated to ensure they accurately reflect the complexities of innovation and investment in the sector. Through this research, we have illustrated that the CBO model does not fully reflect the complex investor decision-making landscape (Table 1). While it was beyond the remit of our interviews and this paper to set out alternative parameterization and modeling structure options, we have demonstrated that the CBO model does not adequately capture the complexity of this ecosystem, and therefore is unlikely to accurately predict the consequences of policy changes on innovation. As the CBO model will continue to inform US drug pricing policy, it should be strengthened through ongoing research and stakeholder input- including from investors. Further, there should be clear and transparent dialogue around what can and cannot be captured by such modeling efforts.

## Supplementary Material

qxaf200_Supplementary_Data
