# Supplementary material for: Understanding biopharmaceutical investment decision-making: how does Congressional Budget Office's model compare to investor insights?
Source: Health Aff Sch. 2025 Oct 23;3(11):qxaf200. doi: 10.1093/haschl/qxaf200 (PMC12602863; doi:10.1093/haschl/qxaf200)
Supplement: qxaf200_Supplementary_Data [file qxaf200_supplementary_data.zip › Supplement 1_ OHE Interview Guide.pdf]

OHE

11. June 2024 / Version 2.0

CONFIDENTIAL

THE BIOPHARMACEUTICAL INNOVATION ECOSYSTEM: BETTER CHARACTERISING R&D INVESTMENT DECISIONS

We are conducting around 20 interviews across different stakeholder groups including large and small biopharmaceutical companies, early-stage venture-capital companies, later-stage growth capital investors and corporate venture capital investors.

This is a semi-structured interview and below you will find the general interview structure and some of the questions that we will address. We may skip some questions and/or ask additional and more detailed questions as relevant to your expertise.

The interview will last approximately one hour. With your permission, the interviews will be recorded for note-taking purposes and recordings will be deleted when notes have been collated.

Interview – Question Guide

Understanding your professional role and business model

- Please briefly describe your company, your role, and how it fits within the landscape of innovation and development
    - At what stage(s) do you invest?
    - Do you invest at the product- or company- level?
    - How much capital do you deploy and where does your capital come from?
    - How mobile is capital? Who are you competing with for capital?

Understanding investment decisions

Costs

- From your perspective, how have R&D costs evolved over time?
    - How does clinical trial duration impact decision-making? E.g. if time to market is faster, can you tolerate higher R&D?

Returns

- How is the value of a product under development or product portfolio calculated?
    - What is the role of market size / revenue as compared to the net profit?
- What is the minimum size of the market opportunity you would need to see to invest in a program in the pre-phase II development phase?
- What exit strategy are you pursuing?

General

- Are investment appraisals typically made at the product- or portfolio- level? Please explain and how you spread your risks in each scenario.

- How big does the investment portfolio need to be in order to spread the investment risk sufficiently? How does this differ per study phase?

- Do investment appraisals typically vary by therapeutic area or mode of action?
- What typically drives your decisions to disinvest in a project or asset and at what point does this normally happen?
- In their simulation model, CBO characterises investment decisions as moving ahead if the NPV of the investment is positive (even if only marginal). Do you agree with this characterisation?

Understanding attitude to risk

- How would you characterise your risk tolerance and risk-reward requirements?
- How do you spread risks?
- How do you determine the size of your investment?
    - What about follow-on investment and dilution?
- To what extent do losses occur on product portfolios and at what research or development stage do they occur most frequently?
- How does your risk appetite change across different stages of the R&D pipeline?
    - What is the role of sunk costs in on-going development decisions?

Understanding policy impact e.g. Inflation Reduction Act.

- How is your company internalising or modelling the impact of US price regulation (if at all?)
- Do you account for price regulation impacts on competitor products within the market?
- Does the reduction in the protected time horizon (i.e. potential for price negotiation to happen after 9 [small molecules] or 13 [biologics] years) mean you are adjusting your NPV calculations for all products undergoing an investment appraisal?
- Do you expect generic /biosimilar markets to be impacted?
- Are small molecules being impacted more than large molecules or advanced therapies?
- Have you already seen a significant impact of the IRA on your R&D/investment budget? Why or why not?
- Has the market already reacted to IRA, e.g. capital mobilized to other sectors?
- Is there anyone else you can suggest we talk to?
